# Molecular dynamics simulations to explore the binding mode between the amyloid-β protein precursor (APP) and adaptor protein Mint2

**DOI:** 10.1038/s41598-024-58584-9

**Published:** 2024-04-04

**Authors:** Min Wang, Kaifeng Liu

**Affiliations:** 1 International Research Centre for Nano Handling and Manufacturing of China, Changchun University of Science and Technology, Changchun, 130022 China; 2 Ministry of Education Key Laboratory for Cross-Scale Micro and Nano Manufacturing, Changchun University of Science and Technology, Changchun, 130022 China; 3 Key Laboratory for Molecular Enzymology and Engineering of Ministry of Education, School of Life Sciences, Jilin University, Changchun, 130012 China

**Keywords:** Amyloid-β (Aβ) protein precursor (APP), Adaptor protein Mint2(Mint2), Molecular dynamics simulations, Comformational changes, MM-PBSA, Biochemistry, Neurochemistry, Proteins

## Abstract

Alzheimer's disease (AD) presents a significant challenge in neurodegenerative disease management, with limited therapeutic options available for its prevention and treatment. At the heart of AD pathogenesis is the amyloid-β (Aβ) protein precursor (APP), with the interaction between APP and the adaptor protein Mint2 being crucial. Despite previous explorations into the APP-Mint2 interaction, the dynamic regulatory mechanisms by which Mint2 modulates APP binding remain poorly understood. This study undertakes molecular dynamics simulations across four distinct systems—free Mint2, Mint2 bound to APP, a mutant form of Mint2, and the mutant form bound to APP—over an extensive 400 ns timeframe. Our findings reveal that the mutant Mint2 experiences significant secondary structural transformations, notably the formation of an α-helix in residues S55-K65 upon APP binding, within the 400 ns simulation period. Additionally, we observed a reduction in the active pocket size of the mutant Mint2 compared to its wild-type counterpart, enhancing its APP binding affinity. These insights hold promise for guiding the development of novel inhibitors targeting the Mints family, potentially paving the way for new therapeutic strategies in AD prevention and treatment.

## Introduction

Alzheimer’s disease (AD) is a chronic degenerative disease. However, few drugs can prevent and treat AD. The etiology of AD is complex, involving genetic and environmental factors. There are many hypotheses on the etiology of AD, including the cholinergic hypothesis^1–3^, abnormal modification of tau protein hypothesis^4–6^, mitochondrial cascade hypothesis^7^, neurovascular hypothesis^8–10^ and inflammation hypothesis^11^. The most widely used and well-known of these is the amyloid hypothesis^12–19^, which suggests that amyloid-β (Aβ) proteins in brain tissue are the main causes of Alzheimer’s disease.

At present, there are three main clinical methods to treat AD, all related to amyloid-β (Aβ) proteins.

Firstly, reduce brain Aβ levels through reducing Aβ production, including β-secretase 1 inhibitors^20–22^ and γ-secretase inhibitors^23–25^. However, most of these inhibitors are terminated in preclinical or clinical trials due to poor selectivity or difficulty in penetrating the blood–brain barrier. The representative drug in this class is Elenbecestat^26–28^, the β-secretase 1 inhibitor, which is used to treat mild cognitive impairment and mild AD. However, Elenbecestat failed to pass the safety review and the development of the product was stopped.

The second category is immunotherapy, including active immunotherapy^29^ and passive immunotherapy^30^. Active immunotherapy enables the body to obtain Aβ immune clearance by inoculation with Aβ antigen, but most of the treatment regimens are terminated due to serious adverse reactions (such as acute meningitis)^30^. Passive immunotherapy, which can eliminate Aβ by injecting human Aβ antibodies, has become a research hotspot because of its ability to avoid severe immune response. This class of drugs is represented by monoclonal antibodies: Gantenerumab^31^, Crenezumab^32^, Ponezumab^33^, GlaxoSmithKline’s (GSK933776A)^34^, Aducanumab^35^, and lecanemab^36^. In particular, Aducanumab has been approved as a new treatment for AD by FDA^16,35^ and is the first new treatment approved to target the underlying disease mechanism of AD. On January 6, 2023, the US FDA granted approval for Leqembi (lecanemab-irmb) through the Accelerated Approval pathway, making it the second new drug to be approved for the treatment of Alzheimer's disease, with the first being Aduhelm. Leqembi is an anti-amyloid (Aβ) protofibrillar antibody used to treat mild cognitive impairment (MCI) and mild Alzheimer's disease (collectively known as early AD) caused by AD. These drugs target the underlying pathophysiological alterations of Alzheimer's disease, representing important advances in ongoing drug development for the effective treatment of this disease^36^.

The third class is Aβ receptor antagonists^37^. These drugs block Aβ downstream pathways so that Aβ that cannot be effectively and safely cleared cannot continue to exert neurotoxic effects^37^. The representative agent in this class is the RAGE antagonist TTP-488^38^.

**Figure 1:**
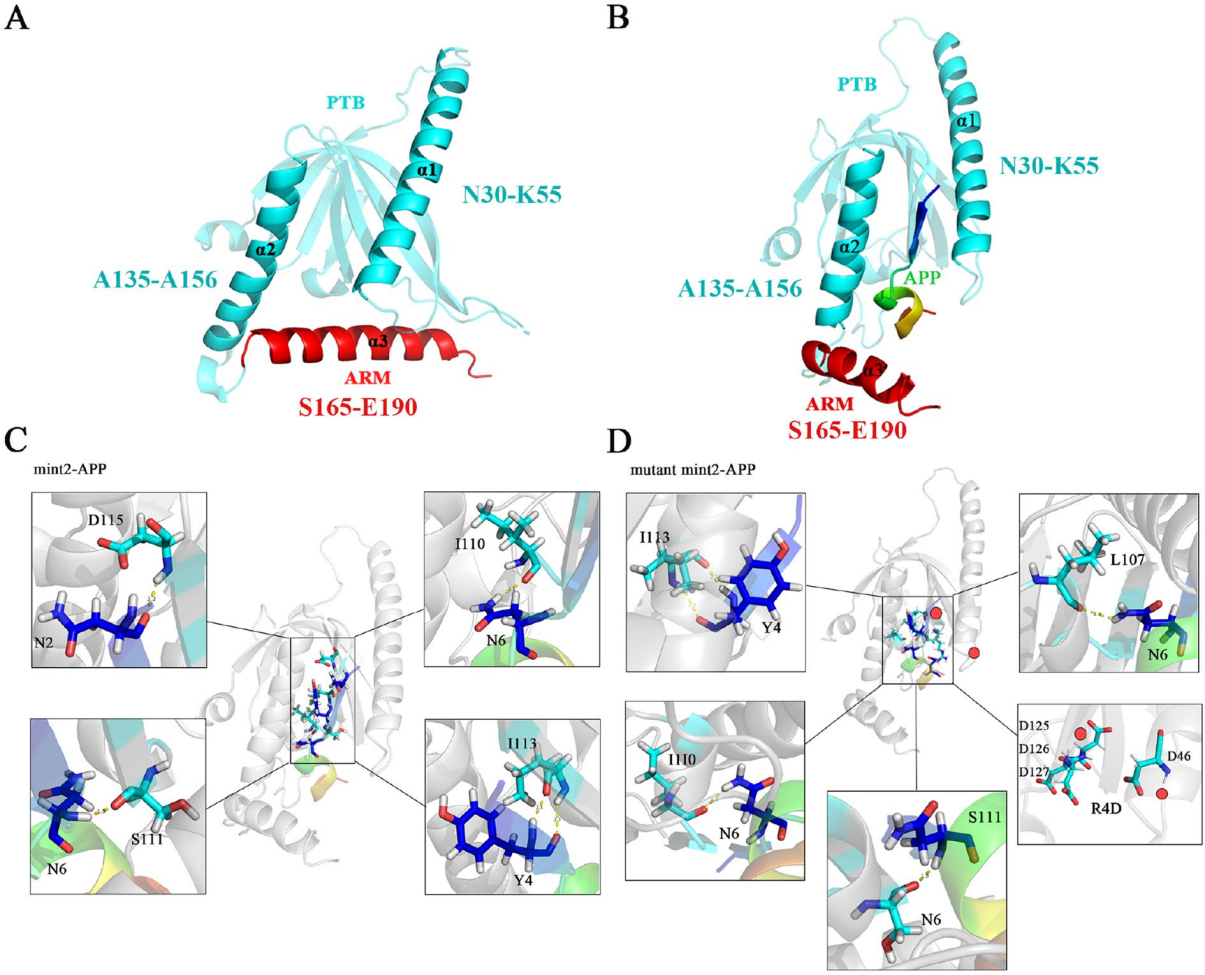
3D structures of PPC. (**A**) close conformation; (**B**) open conformation; (**C**) showed the hydrogen bonds between the WT Mint2 and APP; (**D**) showed the hydrogen bonds between mutant Mint2 (R387D, R473–475D) and APP. The mutated sites were marked with red dots.

Due to the crucial role of Aβ proteins in the pathogenesis of AD, the amyloid-β (Aβ) protein precursor (APP) has also become the focus of research. The regulation of the trafficking and processing of APP depends on the cytosolic proteins that bind to the intracellular tail of APP^39^, including proteins from the Mint family which has three members in mammals: Mint1 (X11/X11a), Mint2 (X11L/X11b), and Mint3 (X11L2/X11g)^40^. Mint family are multi-domain proteins that have a variable N-terminal region and a highly conserved C-terminal region that contains a central PTB domain, a tandem PDZ domain and the very end of C-terminus (named PPC, see Fig. 1A). The phosphotyrosine-binding (PTB) domains found in the Mint family are capable of interacting with the YENPTY motif of APP^40,41^. ARM domain (see Fig. 1B) can block the peptide-binding groove of the PTB domain, regulating APP metabolism. Through these domains, Mints inhibit APP metabolism and thus Aβ generation.

The molecular recognition between APP and adaptor protein Mint2 has previously been addressed^40^. However, the molecular mechanism of Mints’ dynamic regulation of APP binding remains elusive. In this study, four systems (free Mint2, Mint2-APP, mutant Mint2 free, mutant Mint2-APP) were performed with 400 ns molecular dynamics simulations to explore the dynamic changes of Mint2 binding to the APP. Mutants (R387D, R473–475D) were chosen since they can bind to the APP peptide tightly with a K_d_ value < 0.01 μM while wild-type PPC exhibited a K_d_ value of ~ 0.1 μM^42^.

## Results and discussions

### Protein preparation and structure stability

The Mint proteins are multidomain proteins that have a variable N-terminal region and a highly conserved C-terminal region that contains a central PTB domain, a tandem PDZ domain and the very end of the C-terminus (Fig. 1A,B)^39^. Through these domains, Mints mediate the assembly of functional protein complexes. Figure 1C and D showed the hydrogen bonds between the WT Mint2 and APP, mutant Mint2 (R387D, R473–475D) and APP, respectively. There were four hydrogen bonds among Mint2-APP (D115 (refer to Mint2)-N′2 (refer to APP), I110-N′6, I113-Y′4, and I113-Y′4) (Fig. 1C). While There were five hydrogen bonds among mutant Mint2-APP (I113-Y′4, I110-N′6, L107-N′6, R125-N′6 and S111-N′6). The more hydrogen bonds between the complexes, the more stable they are.

MD simulations of all the four systems were performed 5 repeats, respectively.

**Figure 2:**
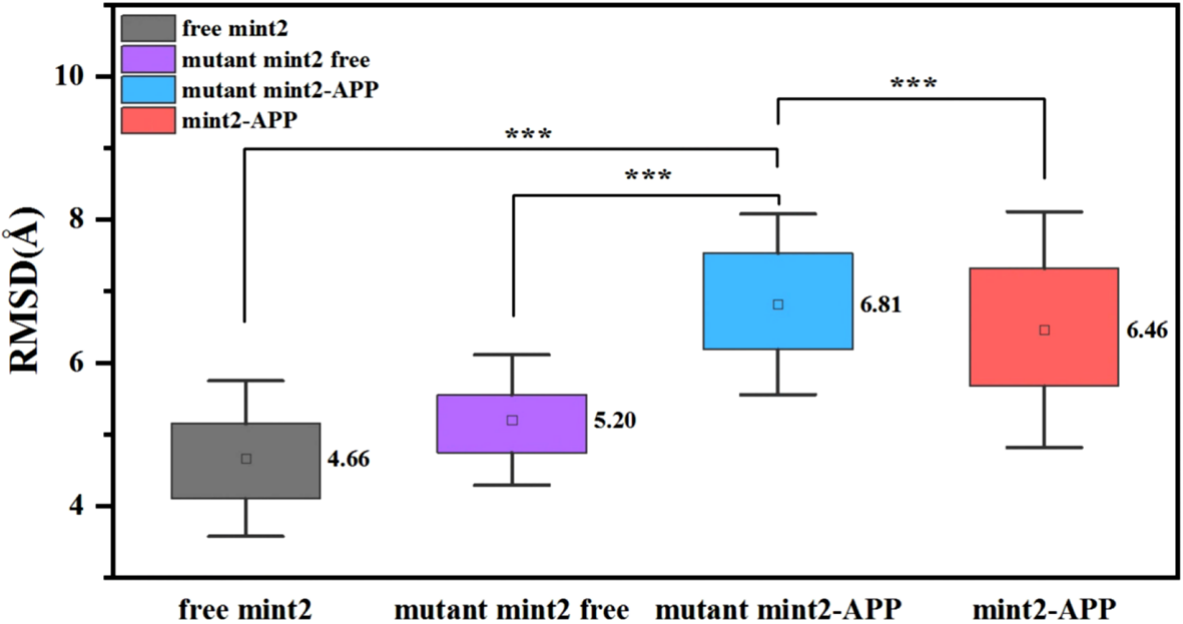
Average RMSD values over 5 replicas and the error bars.

To evaluate the convergence of each system and ensure the reliability of the subsequent sampling strategies, the root-mean-square deviation (RMSD) of C_α_ atoms, was got (See Figure s1 A, C, E, G and I). All simulations got equilibrium after about 300 ns and remained stable during the simulations. And relative frequencies of the RMSDs for 4 systems mainly concentrated in the distribution between 4 and 7 Å (Figure s1 B, D, F, H and J), showing that all four systems had structures that were comparable to their initial structures during the simulations. The average RMSD was calculated for the time interval between 300 and 400 ns as depicted in Fig. 2. Fluctuating RMSD suggested changes in protein structure that were associated with APP binding. A non-parametric test, specifically the Wilcoxon Signed Ranks test, was conducted due to the non-normal distribution of the data. The average RMSD of the five replicates of the mutant Mint2-APP was found to be 6.81 Å, which was significantly higher than the other three systems at a significance level of *p* < 0.001. This means that there is less than a 0.1% hance of obtaining the observed result by chance alone, providing strong evidence that the mutant Mint2-APP system differs significantly from the other three systems in terms of RMSD. The higher RMSD value suggests that the mutant Mint2-APP underwent more conformational changes during the simulation compared to the other systems.

**Figure 3:**
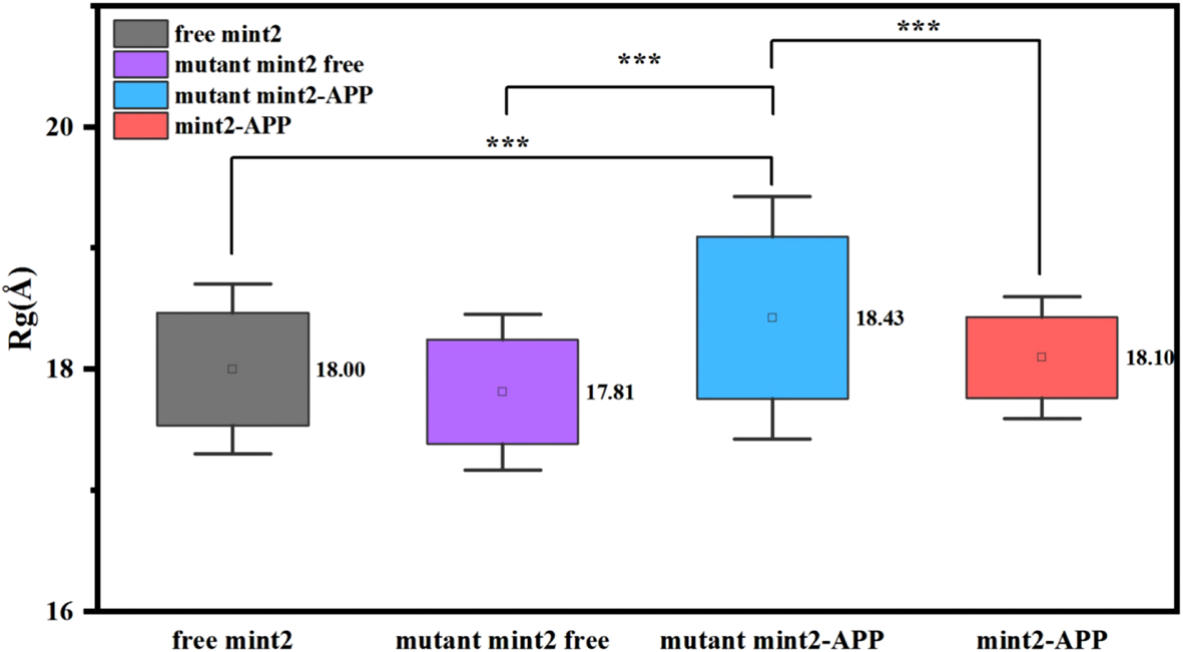
Average Rg values over 5 replicas and the error bars.

As seen in Figure s2 B, D, F, H, and J, the Rg values of mutant Mint2-APP were higher compared to Mint2-APP in all five replicas. The mean Rg values were calculated for a time interval of 300–400 ns, as illustrated in Fig. 3. Due to the non-normal distribution of the data, a non-parametric test, namely the Wilcoxon Signed Ranks test, was conducted. The results showed that the average Rg value for the mutant Mint2-APP was 18.43 Å, which was significantly larger than the other three systems at a significance level of *p* < 0.001. This indicates that the observed difference between the mutant Mint2-APP system and the other systems in terms of Rg is unlikely to have occurred by chance. Taken together, these results suggest that a conformational change occurred in the mutant Mint2-APP system after the introduction of mutations, the mutations have an impact on the overall shape and size of the protein.

**Figure 4:**
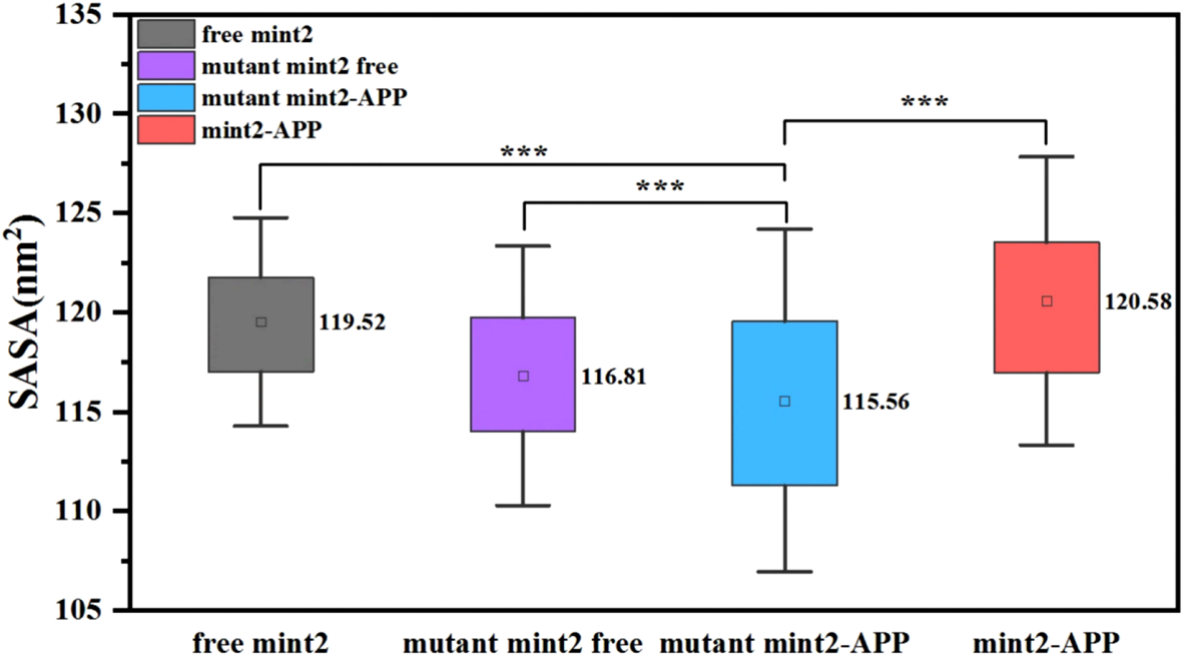
Average SASA values over 5 replicas and the error bars.

From Figure s3, it can be seen that the SASA value of free protein is stable around 110–130 nm^2^ after 200 ns. Compared with the Mint2-APP, the SASA values of the mutant Mint2-APP were decreased in 5 replicas (Figure s3 B, D, F, H and J). And from Fig. 4, the average SASA value of mutant Mint2-APP was found to be 115.56 nm^2^, the Wilcoxon Signed Ranks test was conducted and revealed that this value was significantly smaller than the other three systems at a significance level of *p* < 0.001, indicating that the hydrophilicity of the protein was reduced due to the mutations. These observations suggest that the introduced mutations have an impact on the surface properties of the protein, which may affect its interactions with other molecules such as APP.

To sum up, four systems with 5 replicas were all stable after 400 ns MD simulations and thence can be used for further study.

### Conformational changes during MD simulations

To compare the difference between the flexibility of APP and WT Mint2 as well as the influence of APP on mutant Mint2, root-mean-square fluctuations (RMSF) of the Cα atom were calculated (Figure s4A–J). The largest RMSF change for Mint2-APP and mutant Mint2-APP complexes take place on residues 40–75. The residues 40–75 in Mint2-APP exhibit low RMSF, whereas the RMSFs are increased in mutant Mint2-APP (Figure s4B, D, F, H and J). The flexibility of mutant Mint2-APP is larger than that of WT Mint2-APP. The results imply that the mutated residues may affect the interaction between mutant Mint2 and APP. The difference in the flexibility of the above residues 40–75 may affect the structural fluctuation of WT and mutant Mint2 of APP binding, resulting in different affinity of APP to WT and mutant Mint2. We calculated the mean RMSF value and its standard error for residues 40–75 in the four systems of five replicas. A student's t-test was then applied to the data, which revealed significant differences between the mutant Mint2-APP and Mint2-APP at a significance level of *p* < 0.005.

Our results show that similar RMSF values were observed for key regions across all five replicate experiments. Specifically, in the mutant Mint2-APP, the RMSF of residues 40–75 was consistently higher than that in the WT Mint2-APP in all five replicas, indicating a higher movement intensity for these residues in the mutant Mint2-APP compared to the WT Mint2-APP. These findings suggest that our results are reliable and consistent across all five replicas.

**Figure 5:**
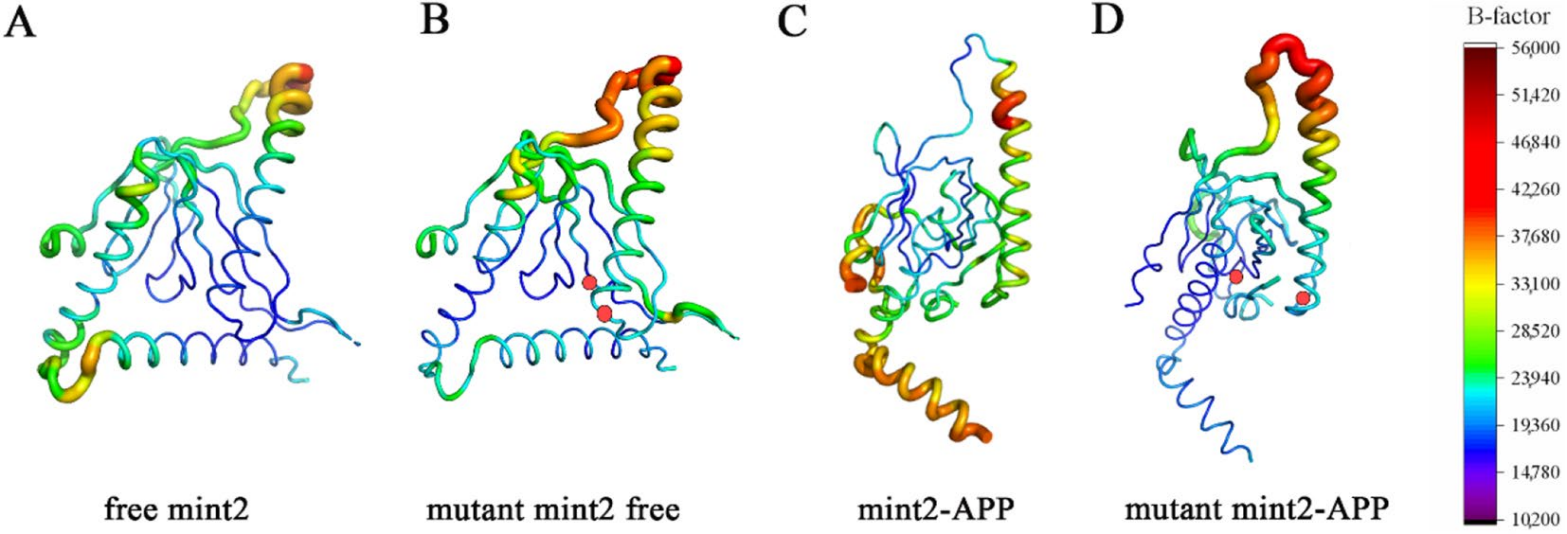
B-factor of four systems. (**A**) Free Mint2. (**B**) Mutant free Mint2. (**C**) Mint2-APP. (**D**) Mutant Mint2-APP. Thicker region indicate a higher B-factor value. The mutated sites were indicated with red dots.

Isotropic temperature factor (B-factor) is a factor that can be applied to each atom, which describes the degree of dispersion of the electron density. Theoretically, the B-factor indicates the static or dynamic flexibility of the atom, and is used to quantify the level of thermal motion, a measure of the uncertainty of the atomic positions in the crystal structure. The average B-factor of five replicas has been calculated to further analyze residual atomic flexibility, with the results displayed in Fig. 5A–D. We can see that the trend of the B-factor is consistent with the RMSF of individual residues (residues 40–75).

The research on the secondary structure of proteins is an indispensable part of the process of MD simulations. The probabilities in Table 1 include data averaged over the five replicate 400 ns experiments and the secondary structure changes can be considered reliable. As seen from Table 2, the average probability of the residues 55–65 domain of mutant Mint2-APP forming α-helices remains steadily higher. While in the WT Mint2-APP, the α-helices in this part dropped and sharply formed loops. The increased helix probability in the mutant may help the substrate slide into the tunnel.

**Table 1:** α-helix probabilities of mutant Mint2-APP and Mint2-APP.

Mu Res	Alpha	Wt Res	Alpha
55	0.97 ± 0.02	55	0.35 ± 0.12
56	0.94 ± 0.03	56	0.31 ± 0.06
57	0.93 ± 0.05	57	0.21 ± 0.12
58	0.97 ± 0.02	58	0.22 ± 0.06
59	0.95 ± 0.01	59	0.15 ± 0.04
60	0.92 ± 0.03	60	0.16 ± 0.07
61	0.89 ± 0.07	61	0.20 ± 0.11
62	0.86 ± 0.03	62	0.15 ± 0.06
63	0.82 ± 0.09	63	0.13 ± 0.03
64	0.43 ± 0.13	64	0.05 ± 0.04
65	0.32 ± 0.04	65	0.03 ± 0.02

**Table 2:** Probabilities of principle component.

Protein	Principle Component	Probability (%)
Mint2-APP	PC1	56.73 ± 7.21
	PC2	19.63 ± 4.33
Mutant Mint2-APP	PC1	71.28 ± 8.18
	PC2	11.06 ± 3.75

**Figure 6:**
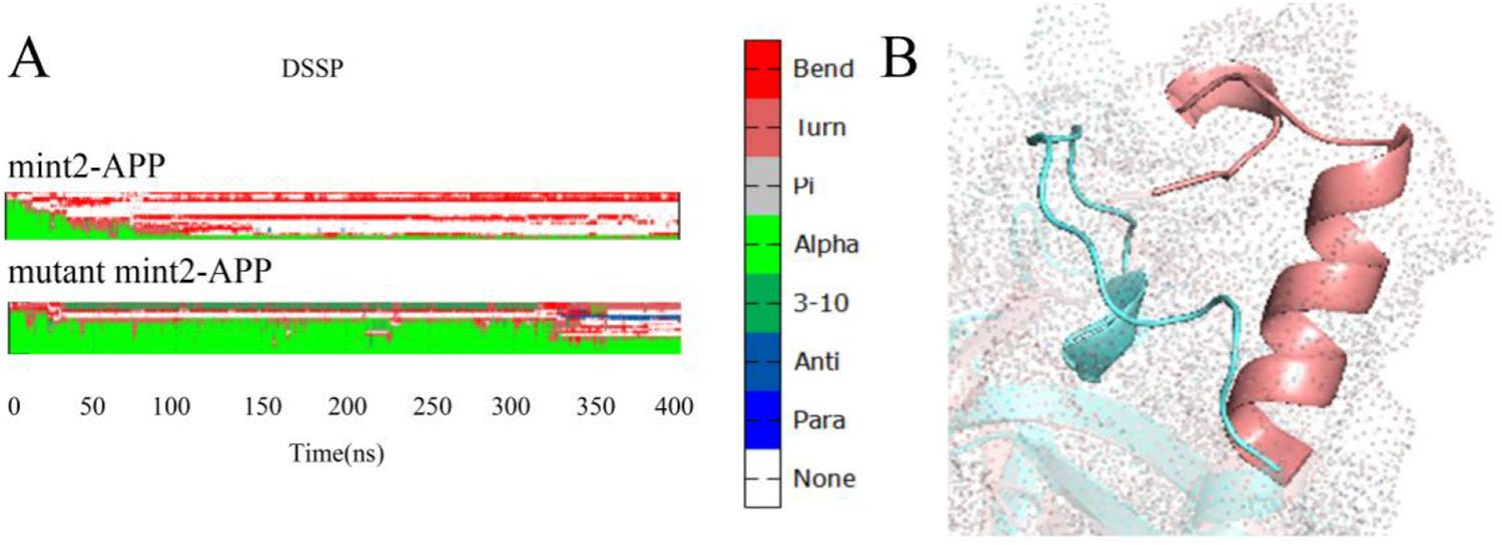
(**A**) Dictionary of secondary structures of mutant Mint2-APP and Mint2-APP. (**B**) Conformation change of S55–D75. Mutant Mint2-APP was shown in pink, while Mint2-APP was shown in cyan.

Our analysis of the Mint2-APP and mutant Mint2-APP systems revealed representative changes in the secondary structure of residues 55–75 in the domain, as shown in Fig. 6A. These representative conformations were obtained through cluster analysis. Figure 6B displays the structure of residues S55-D75 in the representative conformations, highlighting that in the WT Mint2-APP system, the α-helices formed loops. This suggests that the binding of Mint2 to APP in the WT system results in conformational changes in this region, ultimately leading to the formation of these loops. Conversely, in the mutant Mint2-APP system, the α-helice appear to maintain the structure without forming loops, indicating that the introduced mutations may have an impact on the conformational changes observed in this region. DSSP for entire protein with residue number on Y axis was in Fig. s5.

### PCA and hydrogen bonds

Principal component analysis (PCA) was used on 400 ns MD trajectories to determine if the conformational changes were stable. Table 2 listed the averaged probabilities of PC1 and PC2 of the two systems that were got by PCA. PC1 and PC2 accounted for more than 70%, indicating the reliability of results.

**Figure 7:**
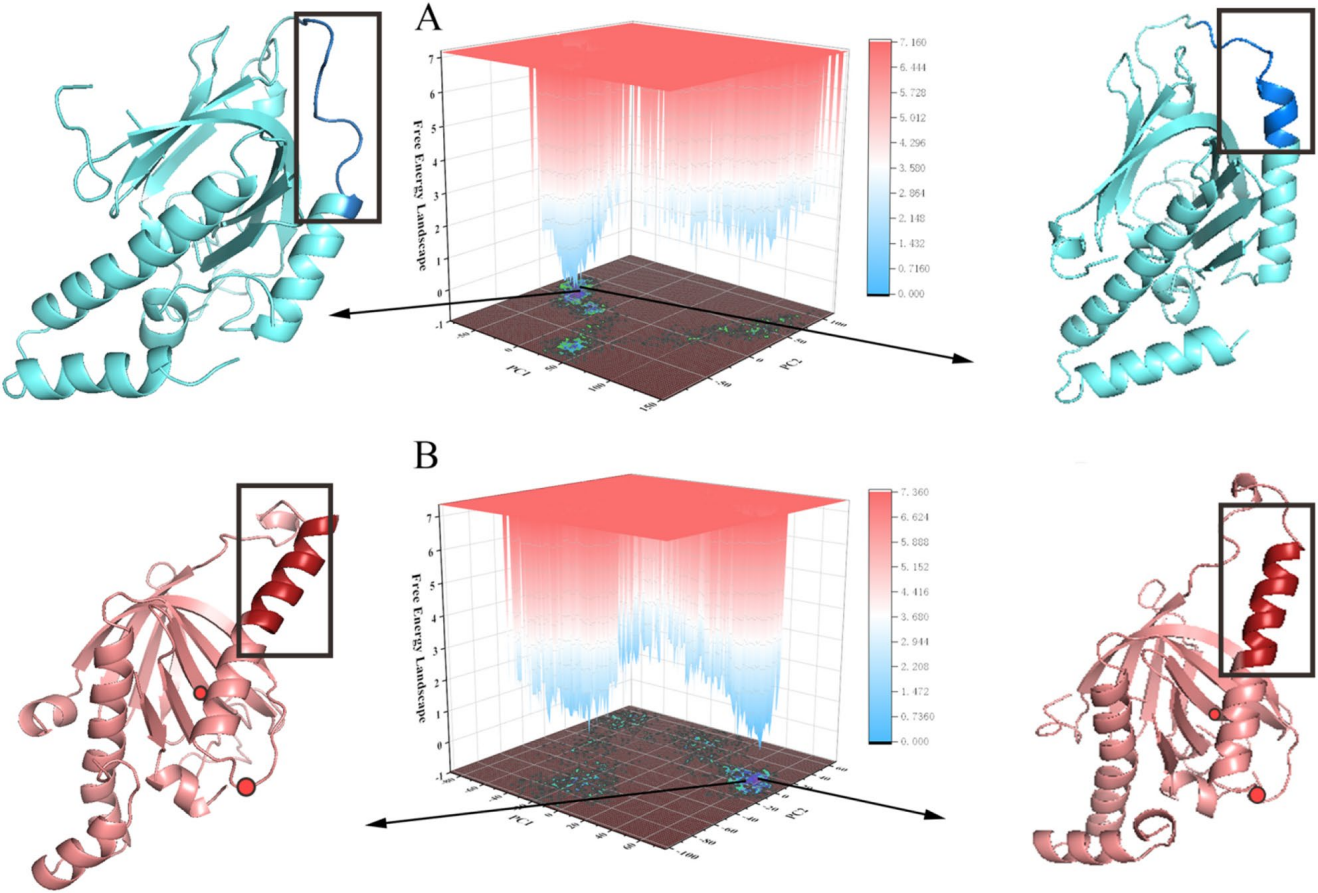
Free energy landscape created by projecting the principle components for (**A**) Mint2-APP; (**B**) mutant Mint2-APP.The conformational changes are showed in black rectangles. The mutated sites were indicated with red dots.

A representative free energy landscape (FEL) which most close to the average data in Table 3 is drawn in Fig. 7A and B. The conformations found in the blue area are more stable and have lower energy states than those found in the red area. The helix (residues 40–50) in mutant Mint2-APP and WT Mint2-APP were highlighted. The structures of the two most stable conformations of the two systems revealed that the conformational changes in the α helix existed between mutant Mint2-APP and WT Mint2-APP. This finding is consistent with the previous analysis. Therefore, the conformational changes were continuous and stable, and the previous analysis is reliable.

**Table 3:** The probability of H-bond between APP and mutant Mint2.

Acceptor	Donor	Probability (%)	Average distance	Average angle
Mint2 ILE_113@O	APP TYR_4@N	67.32 ± 5.56	2.35 ± 0.11	157.81 ± 0.24
Mint2 ILE_110@O	APP ASN_6@ND2	61.44 ± 3.29	2.84 ± 0.06	158.98 ± 0.34
Mint2 SER_111@O	APP ASN_6@N	49.11 ± 2.34	2.88 ± 0.06	163.59 ± 0.45
APP ASN_2@O	Mint2 ASP_115@N	39.3 ± 5.13	2.92 ± 0.09	160.57 ± 0.33
APP GLU_5@O	Mint2 ASN_201@ND2	38.3 ± 3.12	2.85 ± 0.04	157.78 ± 0.13
APP TYR_4@O	Mint2 ILE_113@N	32.21 ± 4.96	2.95 ± 0.14	155.01 ± 0.29
Mint2 ASP_115@OD1	APP ASN_2@N	24.59 ± 2.33	2.86 ± 0.07	153.43 ± 0.37
APP GLU_5@OE1	Mint2 ARG_46@NE	24.45 ± 4.32	2.87 ± 0.08	154.54 ± 0.19
APP GLU_5@OE1	Mint2 ARG_46@NH2	21.85 ± 2.27	2.78 ± 0.09	151.85 ± 0.26

To further compare the inhibitory activities of APP to WT Mint2 and mutant Mint2, we calculated the hydrogen bond probability between APP to WT Mint2 and mutant Mint2 during 400 ns in 5 replicas. The donor and acceptor of the hydrogen bond and the averaged probability of the hydrogen bonds in the 400 ns simulation are listed in Tables 3 and 4. To attain more accurate statistical results, we selected only the hydrogen bonds with a probability greater than 20%. The comparison of these tables shows that the number of hydrogen bonds differed in the two systems. Mutant Mint2-APP was lower than that of WT Mint2-APP. This demonstrates the hydrophobic force may be the reason for the strong binding between APP and mutant Mint2.

**Table 4:** The probability of H-bond between APP and Mint2.

Acceptor	Donor	Probability (%)	Average distance	Average angle
Mint2 ILE_113@O	APP TYR_4@N	78.84 ± 4.84	2.87 ± 0.15	157.34 ± 0.44
Mint2 ILE_110@O	APP ASN_6@ND2	66.91 ± 3.18	2.84 ± 0.09	160.09 ± 0.19
APP ASN_2@O	Mint2 TYR_112@OH	51.75 ± 1.20	2.78 ± 0.06	155.97 ± 0.37
APP GLU_13@OE1	Mint2 ARG_108@NH2	48.95 ± 1.79	2.78 ± 0.13	158.02 ± 0.12
APP GLU_13@O	Mint2 ARG_125@NH1	41.83 ± 3.83	2.89 ± 0.11	153.33 ± 0.24
Mint2 ASP_115@O	APP ASN_2@ND2	38.83 ± 0.76	2.87 ± 0.08	162.57 ± 0.11
APP GLU_13@OE2	Mint2 ARG_108@NH2	37.95 ± 4.35	2.79 ± 0.12	156.01 ± 0.69
APP GLN_14@OXT	Mint2 ARG_39@NH2	36.97 ± 3.21	2.83 ± 0.07	153.18 ± 0.13
APP GLN_14@O	Mint2 ARG_39@NH2	36.36 ± 3.93	2.84 ± 0.14	153.29 ± 0.17
Mint2 SER_111@O	APP ASN_6@N	33.45 ± 2.44	2.91 ± 0.07	161.75 ± 0.32
APP GLN_14@OE1	Mint2 ASN_40@N	34.32 ± 3.23	2.87 ± 0.11	158.82 ± 0.21
APP GLN_14@O	Mint2 ARG_39@NE	31.15 ± 2.92	2.85 ± 0.06	155.07 ± 0.36
APP GLN_14@OXT	Mint2 ARG_39@NE	28.55 ± 3.12	2.83 ± 0.08	153.58 ± 0.43
APP GLN_14@O	Mint2 THR_139@OG1	27.25 ± 1.30	2.68 ± 0.06	165.13 ± 0.08
APP TYR_4@O	Mint2 ILE_113@N	26.85 ± 3.55	2.92 ± 0.12	154.68 ± 0.28
APP GLU_13@OE2	Mint2 ARG_108@NE	26.42 ± 4.73	2.82 ± 0.23	154.81 ± 0.16
APP GLN_1@OE1	Mint2 ASN_44@ND2	25.95 ± 4.08	2.79 ± 0.17	161.64 ± 0.48
APP GLN_14@O	Mint2 THR_140@OG1	21.78 ± 2.11	2.73 ± 0.11	164.22 ± 0.19

### MM-PBSA calculation

As shown in Table 5, the binding free energies of APP to WT Mint2 are higher by 6.93 kcal/mol than those of mutant Mint2, indicating that APP has a better affinity for binding to mutant Mint2 than to WT Mint2. Our conclusion that the binding stability of APP to mutant Mint2 is higher than that of APP to WT Mint2 was confirmed by the binding free energy calculations.

**Table 5:** MM-PBSA calculation.

System	Mint2-APP	Mutant Mint2-APP
△G_vdw_	− 114.11 ± 0.56	− 103.38 ± 0.71
△G_ele_	− 427.55 ± 2.84	− 510.05 ± 5.10
△G_polar_	− 86.50 ± 0.34	− 77.05 ± 0.43
△G_nonpolar_	626.09 ± 2.34	681.48 ± 4.10
△G_gas_	− 541.66 ± 2.80	− 613.43 ± 4.98
△G_solv_	539.58 ± 2.68	604.43 ± 4.53
△G_total_	− 2.07 ± 1.05	− 9.01 ± 1.12

△G_gas_ = △G_vdw_ + △G_ele_.

△G_solv_ = △G_polar_ + △G_nonpolar_.

△G_total_ = △G_gas_ + △G_solv_.

### Active site cavity volume analysis

**Figure 8:**
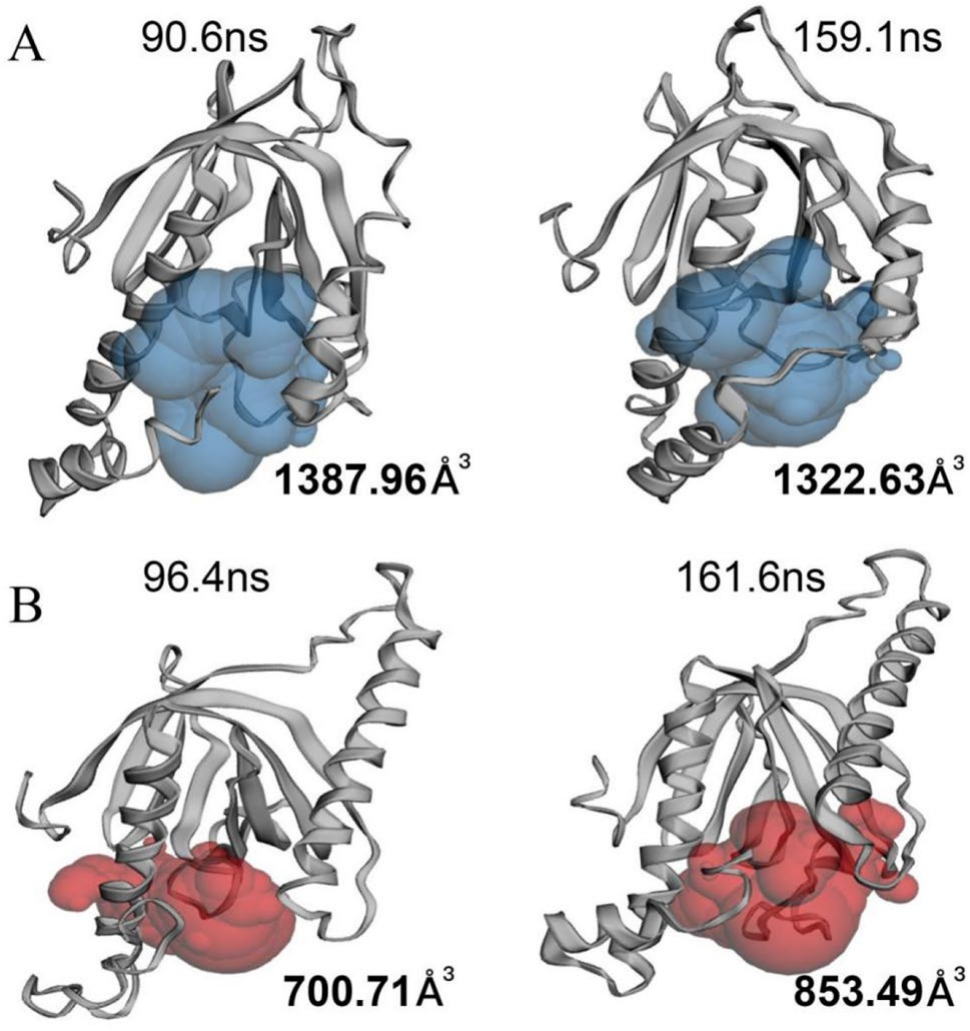
Active site cavity volume of Mint2-APP system and mutant Mint2-APP system. The corresponding time of representative structures were displayed above, and cavity volumes were showed below. The time of occurrence of conformation represented by the cluster is labeled (**A**) Mint2 open. (**B**) mutant Mint2 open. Mutant Mint2′s active pocket becomes smaller.

To investigate whether mutation affected the conformational change in the active site of Mint2, the active site cavity volume of two representative protein structures obtained from cluster analysis for two systems was calculated using the online server CASTp. The cavity volume of Mint2 representative conformations were 1387.96 and 1322.63 Å^3^ respectively (Fig. 8A). Moreover, the mutant protein cavity capacity was significantly reduced, falling to 700.71 and 853.49 Å^3^ (Fig. 8B). Compared to the Mint2 system, the mutant Mint2-APP system's cavity capacity was smaller.

To sum up, the mutant Mint2's active pocket shrinks in comparison to WT Mint2, which is advantageous for APP binding to the enzyme.

## Methods

### System preparation

The 3D Structures of Mint2 (PDB code: 3SUZ)^42^ and Mint2-APP (PDB code: 3SV1)^42^ were obtained from the protein data bank (https://www1.rcsb.org). The water and ligand in the protein were firstly removed using Chimera Software 1.16 (developed by the Resource for Biocomputing, Visualization, and Informatics at the University of California, San Francisco)^43^, and the Modeller plugin was used to model all the missing structures. The mutants (R387D, R473–475D) were constructed using Discovery Studio. After renumbering, G363–A565 were renumbered as G15–A217, and the mutated residues were R39D, R125-127D in our systems.

### Molecular dynamics simulations

In this experiment, Amber 16 software^44,45^ was used to simulate the reaction systems of free Mint2, Mint2-APP, mutant Mint2 free, and mutant Mint2-APP with four 400 ns molecular dynamics simulations (Table 6 contained the details for each system). The Amber FF99SB force field^46,47^ were used, and the TIP3P water model^48,49^ was added for these systems. To avoid edge effects, periodic boundary conditions were given to the reaction system during the simulation duration. The distance between the solute surface and the box was set to 12 Å. Because the charge in the initial reaction system is not zero, it was necessary to add Na^+^ to the system in the initial stage of reaction simulation.

**Table 6:** The information of 4 systems.

Systems	Free Mint2 (G363–A565)	Mint2-APP (G363–A565)	Mutant Mint2 free (G363–A565)	Mutant Mint2-APP (G363–A565)
Residues	190	204(1-14APP, 15-204Mint2)	190	204(1-14APP, 15-204Mint2)
Na^+^	2	3	3	4
WAT	13,243	13,246	14,475	14,476
Volume (nm^3^)	530.202	530.202	530.202	530.202

After the systems were constructed, energy minimization for the four systems was carried out to eliminate atomic collisions in the initial structures. The whole process is divided into two parts, the steepest descent and the conjugate gradient method with 500 steps respectively. The initial structures of the systems were stable after energy minimization, and the reaction time of 50 ps was used to raise the temperature of the simulated reaction from 0 to 300 K. After heating, the simulated systems were then treated with 50 ps of reaction time for density equilibration. Finally, the simulated systems were equilibrated with a constant pressure operation under NPT ensemble, with a constant pressure balance of 500 ps at 300 K. Constant pressure equilibration was the last step of system equilibration. After all, the thermodynamic parameters were stabilized, and 400 ns molecular dynamics simulations were carried out for the four simulated systems. And the experimental data collection interval was set at 1 fs for each system. The storage interval is 2 ps/interval and the total record structure is 10,000 frames. The data were kept for further study and analysis. Each MD simulation was performed five times.

### Trajectory analysis

The CPPTRAJ module of Amber16 was used for the trajectory analysis, which included calculations for the RMSD, RMSF, R_g_, SASA, dictionary of secondary structures, and hydrogen bond analysis^50^, and the error bars were calculated using Origin. K-means clustering was also performed using CPPTRAJ, ten representative structures were obtained. The script of structural analysis can be found in the Supporting materials.

### Principal component analysis and free energy landscape

Principal component analysis (PCA)^51^ is a widely used dimensionality reduction method that describes the coordinated motion of the entire protein. In this method, MD trajectories were used to construct covariance matrices of atomic coordinates. By diagonalizing the covariance matrix, eigenvectors and eigenvalues characterizing the coordinated motion of proteins can be obtained. The eigenvectors describe the direction of motion, and the corresponding eigenvalues represented the amplitude of motion along these eigenvectors. The first few major components were generally thought to represent functionally important movements in proteins. PCA extracted relevant fluctuations from molecular dynamics simulation trajectories by using the covariance matrix of all conformations relative to the covariance matrix of the average structure to reduce dimensionality. Based on the diagonalization of the covariance matrix, PCA gives the orthogonal eigenvectors and their corresponding eigenvalues. The element C_ij_ of the matrix is defined as:

$$C_{ij} = \frac{{\left\langle {\Delta ri \cdot \Delta rj} \right\rangle }}{{(\left\langle {\Delta ri^{2} } \right\rangle \cdot \left\langle {\Delta rj^{2} } \right\rangle )^{1/2} }}$$

Here, Δ_ri_ (Δ_rj_) is the displacement vector corresponding to the i-th (j-th) atom of the system and represents the ensemble average. The eigenvectors of the matrix represent the direction of coordinated motion. The eigenvalues are the magnitude of the motion in one direction. In general, the first few principal components (PCs) describe the most important movements associated with the functional movements of the biomolecular system.

The free energy landscapes (FELs) are often used to find the dominant conformation, since the free-energy minimum usually represents the conformational ensemble in the steady state, while the free-energy barrier represents the transient state. FEL was constructed based on the PCA data. FEL can be expressed as:

$$\Delta G\left( X \right) = - KBT\ln P(X)$$

Here, K_B_ is Boltzmann’s constant, T is the temperature, and P (X) is the probability distribution along the reaction coordinates. In this study, we calculated FEL to identify the major conformational states with relatively low energy.

### MM—PBSA calculation

The MM-PBSA method as applied to small molecule binding is an end-point method estimating the binding free-energy difference between the protein–ligand complex^52–55^. The single-trajectory approach is favored for its straightforward implementation and cancellation of covalent energy errors as conformations for the complex and separated receptor and ligand are based on shared configurations from MD simulations.

MM-PBSA is often used in tandem with the closely related Molecular Mechanics Generalized Born Surface Area (MM-GBSA) approach as both utilize the same set of inputs for the prediction of binding free energies with continum solvation^56–58^:

1 $$\Delta {\text{G}}_{{{\text{bind}}}} = {\text{ G}}_{{{\text{complex}}}} - {\text{ G}}_{{{\text{receptor}}}} - {\text{ G}}_{{{\text{ligand}}}}$$

2 $$\Delta {\text{G}}_{{{\text{bind}}}} = \, \Delta {\text{H }} - {\text{ T}}\Delta {\text{S}}$$

3 $$\Delta {\text{H }} = \, \Delta {\text{E}}_{{{\text{ele}}}} + \, \Delta {\text{E}}_{{{\text{vdW}}}} + \, \Delta {\text{G}}_{{{\text{PB}}}} + \, \Delta {\text{G}}_{{{\text{SA}}}}$$

4 $$\Delta {\text{G}}_{{{\text{SA}}}} = \, \gamma \Delta {\text{SASA }} + \, \beta$$

In our calculation, γ and β were set to 0.00542 kcal mol^−1^/Å^−2^ and 0.92 kcal mol^−1^, respectively. For the ionic strength, a value of 0.1 M was used, and for the dielectric constants of the solvent and the solute, values of 80.0 and 1.0 were used, respectively^59,60^.

## Conclusions

In this study, we used 400 ns molecular dynamics simulation to research four systems (free Mint2, Mint2-APP, mutant Mint2 free, mutant Mint2-APP). The results show that during 400 ns MD simulation, the residues S55–K65 of mutant Mint2 had undergone secondary structure changes and formed alpha-helix after combining with APP. Compared with WT-Mint2, the binding free energy was reduced, indicating that mutation was helpful to enhance the binding of APP. Also, there was less chance that the APP and mutant Mint2 form hydrogen bonds during binding, and the strong binding may be caused by hydrophobic force. Molecular dynamics simulations were used to reveal for the first time the effect of Mint2 mutation on binding to APP and its mechanism. Our results may provide clues for the design of new Mint inhibitors.

### Supplementary Information

Supplementary Information.

## Data Availability

The datasets generated and analysed during the current study are available from the corresponding author on reasonable request.
