# Supplementary material for: Molecular dynamics simulations to explore the binding mode between the amyloid-β protein precursor (APP) and adaptor protein Mint2
Source: Sci Rep. 2024 Apr 4;14:7975. doi: 10.1038/s41598-024-58584-9 (PMC10995209; doi:10.1038/s41598-024-58584-9)
Supplement: Supplementary file 1 — Supplementary Information. [file 41598_2024_58584_MOESM1_ESM.pdf]

Molecular Dynamics Simulations to Explore the Binding Mode between the Amyloid-β protein Precursor (APP) and Adaptor Protein Mint2

Min Wang [1*†], Kaifeng Liu [2†]

1.International Research Centre for Nano Handling and Manufacturing of China, Changchun University of Science and Technology, Changchun 130022, China
2.Ministry of Education Key Laboratory for Cross-Scale Micro and Nano Manufacturing, Changchun University of Science and Technology, Changchun 130022, China
3.Key Laboratory for Molecular Enzymology and Engineering of Ministry of Education, School of Life Sciences, Jilin University, Changchun 130012, China
* Correspondence: minwang20@mails.jlu.edu.cn (M.W.)
† These authors contributed equally to this work.

CPPTRAJ Script:

```
# RMSD, RMSF, Rg, SASA
parm nowat.prmtop
trajin nowat.dcd
rms first mass out rmsd.dat :15-204@CA=
run
atomicfluct byres out rmsf.dat :15-204@CA=
run
radgyr :15-204&!(@H=) out rg.dat mass nomax
run
surf :15-204 out surf.dat
run
# DSSP
parm nowat.prmtop
trajin nowat.dcd
autoimage
secstruct :1-204 out dssp.dat
Run
# B-factor
parm nowat.prmtop
trajin nowat.dcd
atomicfluct out back.apf @C,CA,N byres bfactor
run
# K-means clustering
parm nowat.prmtop
trajin nowat.dcd
strip :Na+,Cl-
cluster c1 kmeans clusters 10 randompoint maxit 500 rms :15-204@C,N,O,CA,CB&!@H= sieve 10 random out cnumvtime.dat summary summary.dat info info.dat cpopvtime cpopvtime.agr normframe repout rep repfmt pdb singlerepout singlerep.nc singlerepfmt netcdf avgout avg avgfmt pdb
run
# PCA
parm nowat.prmtop
trajin nowat.dcd
rms first :15-204&!@H=
average crdset proavy
run
```

```
rms ref proavy :15-204&!@H=
matrix covar name MyMatrix :15-204&!@H=
createcrd CRD1
run
runanalysis diagmatrix MyMatrix vecs 2 name MyEvecs
crdaction CRD1 projection evecs MyEvecs :15-204&!@H= out project.dat beg 1 end 2
quit
# H-Bond
parm nowat.prmtop
trajin nowat.dcd
hbond donormask :1-14@F=,O=,N= acceptormask :15-204@F=,O=,N= out nhb1.dat avgout
avghb1.dat
run
hbond donormask :15-204@F=,O=,N= acceptormask :1-14@F=,O=,N= out nhb2.dat avgout
avghb2.dat
run
```

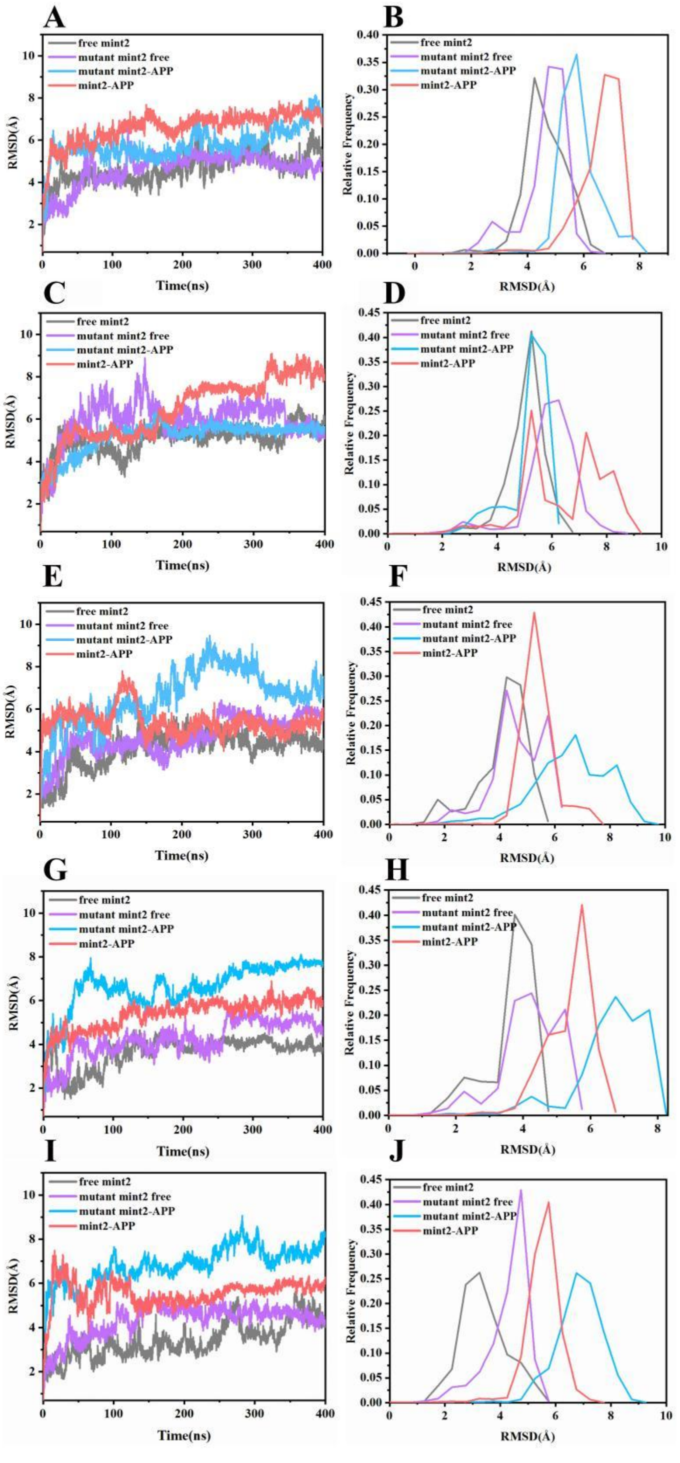

Figure s1. (**A**) The temporal evolution of the RMSDs from their initial structures of 4 systems. (**B**) Relative frequencies of the RMSDs for 4 systems. (**C**) The temporal evolution of the RMSDs from their initial structures of 4 systems in replica 1. (**D**) Relative frequencies of the RMSDs for 4 systems in replica 1. (**E**) The temporal evolution of the RMSDs from their initial structures of 4 systems in replica 2. (**F**) Relative frequencies of the RMSDs for 4 systems in replica 2. (**G**) The temporal evolution of the RMSDs from their initial structures of 4 systems in replica 3. (**H**) Relative frequencies of the RMSDs for 4 systems in replica 3. (**I**) The temporal evolution of the RMSDs from their initial structures of 4 systems in replica 4. (**J**) Relative frequencies of the RMSDs for 4 systems in replica 4.

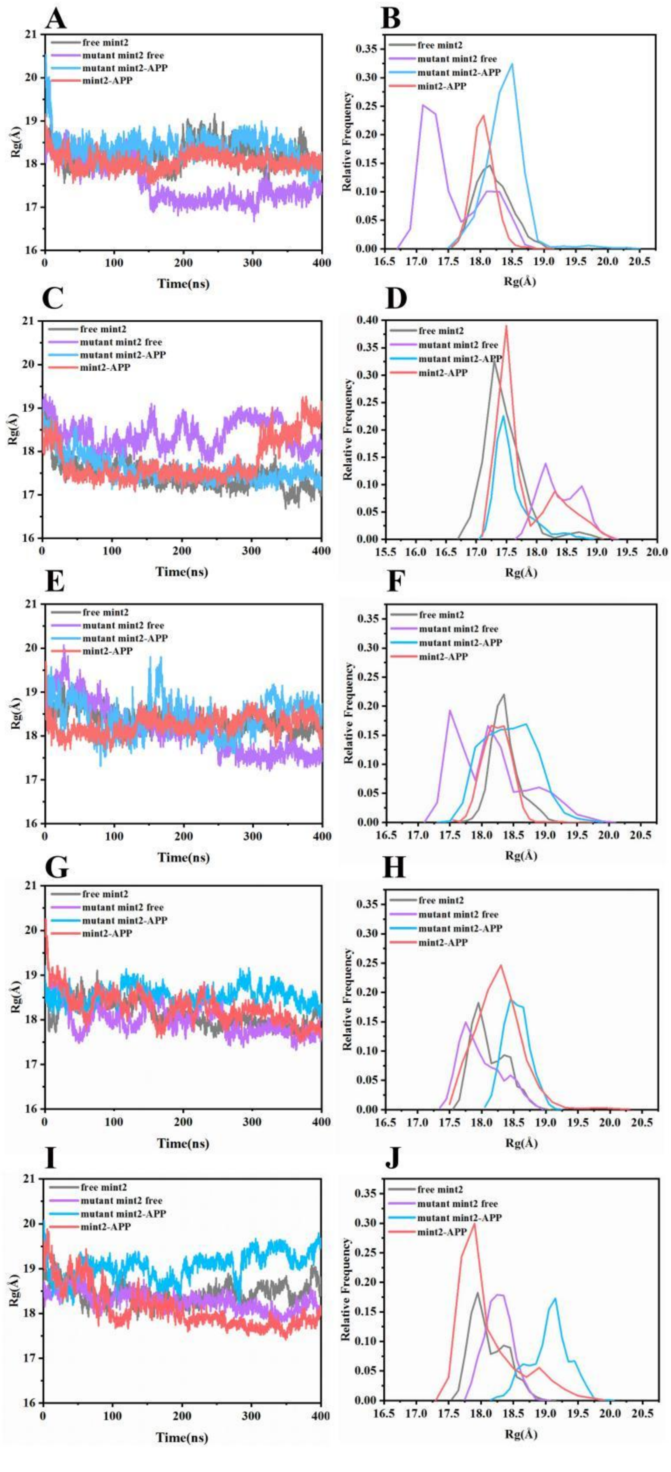

Figure s2. (**A**) The radius of gyration of 4 systems during 400 ns simulation. (**B**) Relative frequencies of the Rg values for 4 systems. (**C**) The radius of gyration of 4 systems during 400 ns simulation in replica 1. (**D**) Relative frequencies of the Rg values for 4 systems in replica 1. (**E**) The radius of gyration of 4 systems during 400 ns simulation in replica 2. (**F**) Relative frequencies of the Rg values for 4 systems in replica 2. (**G**) The radius of gyration of 4 systems during 400 ns simulation in replica 3. (**H**) Relative frequencies of the Rg values for 4 systems in replica 3. (**I**) The radius of gyration of 4 systems during 400 ns simulation in replica 4. (**J**) Relative frequencies of the Rg values for 4 systems in replica 4.

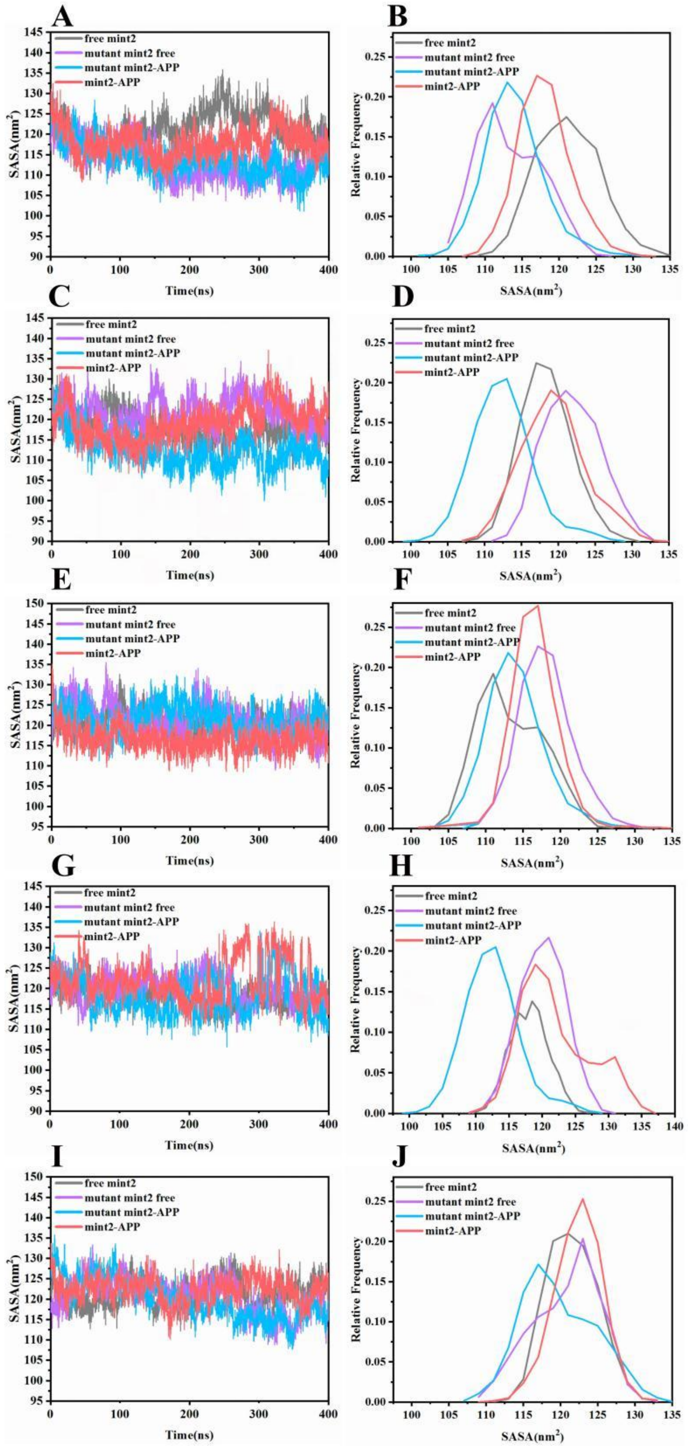

Figure s3. (**A**) SASA values of four systems over 400 ns MD. (**B**) Relative frequencies of the SASA for 4 systems. (**C**) SASA values of four systems over 400 ns MD in replica 1. (**D**) Relative frequencies of the SASA for 4 systems in replica 1. (**E**) SASA values of four systems over 400 ns MD in replica 2. (**F**) Relative frequencies of the SASA for 4 systems in replica 2. (**G**) SASA values of four systems over 400 ns MD in replica 3. (**H**) Relative frequencies of the SASA for 4 systems in replica 3. (**I**) SASA values of four systems over 400 ns MD in replica 4. (**J**) Relative frequencies of the SASA for 4 systems in replica 4.

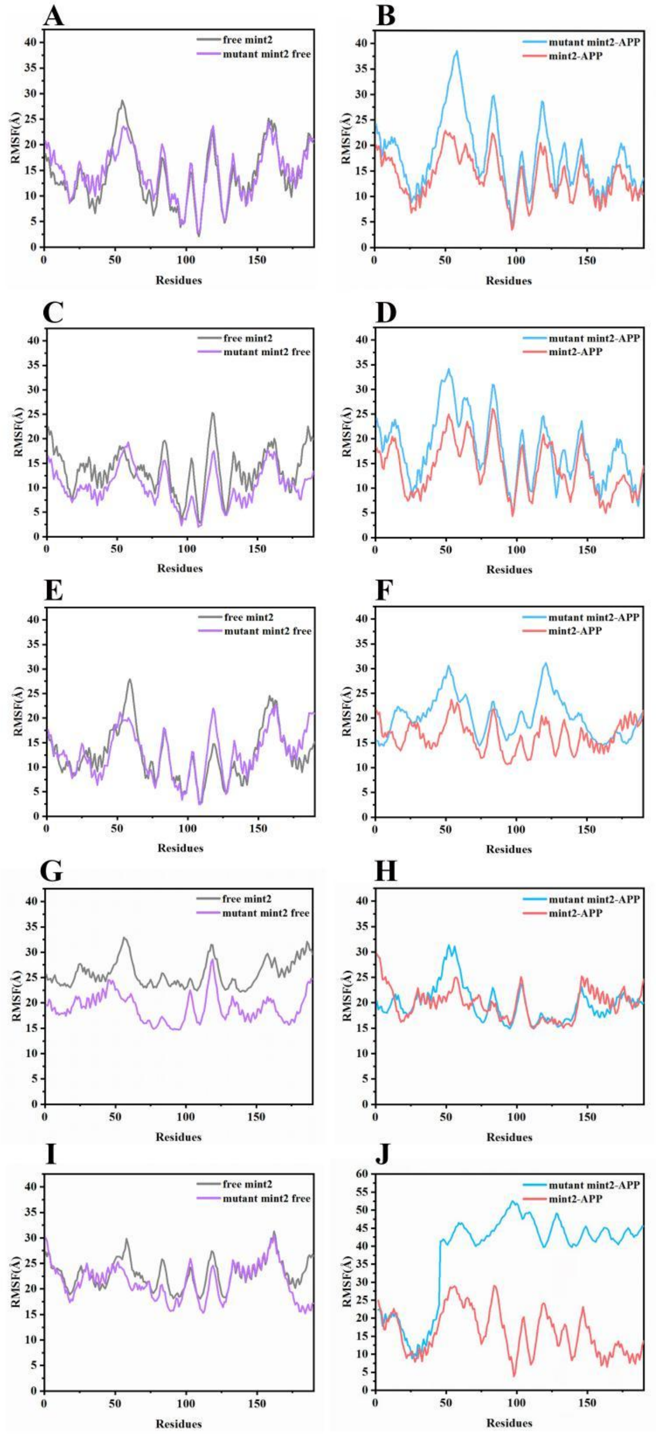

Figure s4. RMSFs of Cα atoms in the four systems: (**A**) free Mint2 and mutant Mint2 free. (**B**) Mint2-APP and mutant Mint2-APP. The major changes in RMSF occur on the residues 40–75 for complexes. (**C**) RMSFs of free Mint2 and mutant Mint2 free in replicate 1. (**D**) RMSFs of Mint2-APP and mutant Mint2-APP in replicate 1. (**E**) RMSFs of free Mint2 and mutant Mint2 free in replicate 2. (**F**) RMSFs of Mint2-APP and mutant Mint2-APP in replicate 2. (**G**) RMSFs of free Mint2 and mutant Mint2 free in replicate 3. (**H**) RMSFs of Mint2-APP and mutant Mint2-APP in replicate 3. (**I**) RMSFs of free Mint2 and mutant Mint2 free in replicate 4. (**J**) RMSFs of Mint2-APP and mutant Mint2-APP in replicate 4

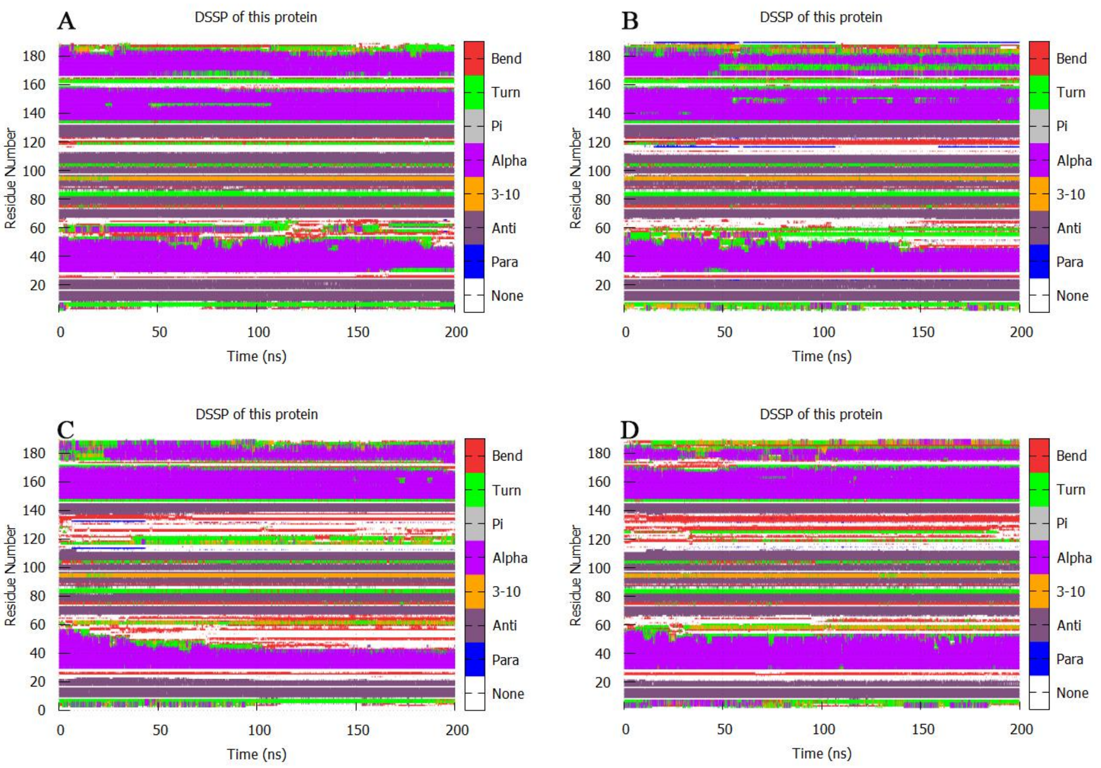

Figure s5. DSSP for entire protein (**A**) Free Mint2. (**B**) Mutant free Mint2. (**C**) Mint2–APP. (**D**) Mutant Mint2–APP.
